# 23-hydroxybetulinic acid induces cell cycle arrest in esophageal cancer cells via the BUB1/STAT3 signaling pathway

**DOI:** 10.3389/fonc.2026.1804674

**Published:** 2026-05-05

**Authors:** Hui Yang, Gao Si, Xi Zhou, Xuejie Song, Haiyang Du, Fuchun Si

**Affiliations:** 1 Traditional Chinese Medicine School, Henan University of Chinese Medicine, Henan Key Laboratory of Traditional Chinese Medicine Syndrome and Prescription in Signaling, Henan International Joint Laboratory of Traditional Chinese Medicine Syndrome and Prescription in Signaling, Zhengzhou, China; 2 Orthopaedic Department, Peking University Third Hospital, Beijing, China; 3 Academy of Chinese Medical Sciences, Henan University of Chinese Medicine, Zhengzhou, China

**Keywords:** 23-Hydroxybetulinic acid, Bub1, cell cycle, esophageal cancer, Stat3 signaling pathway

## Abstract

**Background:**

23-Hydroxybetulinic acid (23-HBA), a key bioactive compound in the traditional Chinese herb *Pulsatilla chinensis*, has garnered substantial scientific interest due to its potent antitumor activity. However, the inhibitory effects of 23-HBA on esophageal cancer growth have not been fully elucidated.

**Methods:**

In this study, EC9706 and KYSE150 cells were treated with 23-hydroxybetulinic acid (23-HBA). Cell viability was assessed using the CCK-8 assay. Cell cycle distribution and apoptosis were analyzed by flow cytometry. Cell migration and invasion abilities were evaluated using wound healing and Transwell invasion assays, respectively. Protein expression levels of BUB1, STAT3, p-STAT3, CCNB1, CDK1, Bcl-2 and Caspase-3 were determined by Western blot analysis. For the *in vivo* study, a nude mouse xenograft model of esophageal carcinoma was established. Tumor-bearing mice were randomly assigned to the following groups: model group, low-dose (15 mg/kg) and high-dose (30 mg/kg) 23-HBA groups, and cisplatin group (4 mg/kg). Body weight and tumor volume were monitored regularly. At the end of the experiment, tumor tissues were collected, weighed, and subjected to histopathological examination via hematoxylin and eosin (HE) staining. Protein expression of BUB1, STAT3, and p-STAT3 in tumor tissues was further analyzed by Western blot and immunohistochemistry (IHC). Meanwhile, the protein expressions of Bcl-2 and Caspase-3 were detected.

**Results:**

23-HBA inhibited the proliferation, migration, and invasion of EC cells, while inducing apoptosis and G2/M phase cell cycle arrest. Treatment with 23-HBA significantly downregulated the expression of p-STAT3, BUB1, CCNB1, CDK1, and Bcl-2 in EC cells, while significantly upregulating the expression of Caspase-3, although STAT3 protein levels remained unchanged. In the xenograft mouse model, 23-HBA treatment led to a significant reduction in tumor volume and weight compared with the model group, accompanied by extensive tumor necrosis. Western blot and immunohistochemical analyses further confirmed that p-STAT3 and BUB1 expression were markedly downregulated in tumor tissues from the 23-HBA-treated group, whereas STAT3 expression did not show significant alterations. The results showed that 23-HBA downregulated Bcl-2 and upregulated Caspase-3 protein expression.

**Conclusion:**

23-HBA inhibits mitosis in esophageal cancer by blocking the BUB1/STAT3 signaling pathway, suggesting its potential as a therapeutic agent for esophageal cancer treatment.

## Introduction

Esophageal carcinoma (EC), one of the most common malignant tumors of the digestive system, is characterized by high incidence, aggressive progression, and unfavorable prognosis, esophageal squamous cell carcinoma and esophageal adenocarcinoma are the two major histological types of esophageal carcinoma, with the former representing approximately 90% of global cases (1). Although therapeutic approaches—including surgery, chemotherapy, radiotherapy, and immunotherapy—have been progressively refined (2), the overall 5-year survival rate remains below 20%, rendering EC one of the most lethal cancers globally (3). Treatment outcomes are further limited by challenges such as acquired drug resistance, variable patient tolerance, and therapy-related adverse effects. Consequently, developing novel agents and therapeutic strategies is essential to improve the clinical management of esophageal cancer.

Traditional Chinese Medicine (TCM), with a history spanning thousands of years in China, has contributed numerous monomeric compounds with demonstrated efficacy in cancer treatment (4, 5). The discovery of active constituents from medicinal plants remains a valuable source for novel therapeutic agents that target diverse pharmacological pathways, including those involved in oncology (6). However, due to the complex composition and multifaceted nature of herbal medicines, elucidating the precise mechanisms of action of their primary constituents and identifying specific therapeutic targets in cancer treatment continue to pose significant challenges.

*Pulsatilla chinensis* (Bai Tou Weng) is a traditional Chinese medicinal herb characterized by a bitter taste and cold property. It has been traditionally used to clear heat, detoxify, and cool blood for the treatment of dysentery (7). Modern pharmacological studies have further confirmed that its bioactive compounds possess notable anti-tumor and immunomodulatory activities (8). 23-Hydroxybetulinic acid (23-HBA), the principal triterpenoid constituent of *Pulsatilla chinensis*, is recognized as a key active component contributing to its anti-tumor properties. Previous studies have shown that 23-HBA can inhibit the growth of various tumor types, including colon, lung, and liver cancers (9–11). Additionally, evidence suggests that 23-HBA may enhance tumor cell sensitivity to chemotherapeutic agents, thereby exerting synergistic anti-tumor effects (12).

Budding uninhibited by benzimidazole 1 (BUB1) is a critical spindle assembly checkpoint protein essential for proper chromosome alignment during mitosis (13). It has been found dysregulated in various human cancers, including breast, pancreatic, gastric, and liver cancer (14, 15). Previous studies have shown that BUB1 facilitates bladder tumorigenesis and progression by modulating STAT3 expression (16). However, the role of BUB1 has not been systematically investigated in EC.

Our previous study revealed that Baitouweng decoction inhibits the growth of esophageal cancer cells via modulation of the BUB1/STAT3 signaling pathway (17). Building on this finding, the present study aimed to further elucidate the mechanism by which 23-HBA, a bioactive compound derived from *Pulsatilla chinensis*, exerts its anti-tumor effects in esophageal cancer. Our results demonstrate that 23-HBA suppresses esophageal cancer progression through BUB1-mediated STAT3 signaling. These findings offer novel mechanistic insights into the therapeutic potential of 23-HBA as a targeted agent for esophageal cancer treatment.

## Materials and methods

### Reagents

23-Hydroxybetulinic acid (Cat.No. B21763, purity≥98%) was purchased from Shanghai Yuanye Bio-Technology Co., Ltd (Shanghai, China). Cisplatin (Cat. No. 232120, purity ≥98%) was purchased from Merck & Co., Inc. (USA).

### Cell culture

Human esophageal squamous cell carcinoma lines EC9706 and KYSE150 were procured from the Henan Key Laboratory of Traditional Chinese Medicine Syndrome and Prescription in Signaling at Henan University of Chinese Medicine. The cells were cultured in 100 mm dishes containing 10 mL of RPMI 1640 medium supplemented with 10% fetal bovine serum (FBS), and maintained at 37 C in a humidified atmosphere with 5% CO_2_.

### Cell proliferation assay

Cell viability was evaluated using the Cell Counting Kit-8 (CCK-8) assay. Briefly, EC9706 and KYSE150 cells (3,000 cells/well) were seeded into 96-well plates and treated with various concentrations of 23-HBA (0, 25, 50, 100, 200, 400, and 800 µg/mL) for 48 hours. Following treatment, CCK-8 reagent was added according to the manufacturer’s instructions, and optical density (OD) at 450 nm was measured using a microplate reader (ThermoFisher Scientific, CA, USA). The inhibition rate was calculated to assess the dose-dependent effect of 23-HBA, and the half-maximal inhibitory concentration (IC_50_) was derived based on the standard curve.

### Cell cycle analysis

Cells were seeded into 100 mm dishes at a density of 1×10^6^ cells per dish. After 24 hours, the cells were treated with varying concentrations of 23-HBA for 48 hours, harvested, and fixed overnight in 70% ethanol at -20 C. The fixed cells were then washed with ice−cold PBS, stained with RNase A (20 µg/mL) and propidium iodide (PI, 200 µg/mL) in the dark for 30 minutes, and subsequently analyzed by flow cytometry (BD Biosciences, USA) to determine cell cycle distribution.

### Cell apoptosis analysis

Cell apoptosis was assessed using an Annexin V-FITC/PI Apoptosis Detection Kit (BD Biosciences, USA). Following 48 hours of treatment under the indicated conditions, cells were washed twice with PBS and resuspended in an appropriate volume of binding buffer. The suspension was then incubated with 10 μL Annexin V-fluorescein isothiocyanate (FITC) in the dark for 15 minutes, followed by the addition of 10 μL PI. Apoptotic rates were immediately analyzed by flow cytometry (BD Biosciences, USA).

### Wound healing assay

A wound-healing assay was conducted to evaluate cell migration. Cells were seeded into 6-well plates at a density of 3×10^5^ cells per well. After 24 hours, a uniform scratch was created in the monolayer using a 200 µL pipette tip. The cells were washed with PBS and then incubated with the designated treatments. Wound closure was monitored and imaged at 0, 24, and 48 hours post-scratch, and the migration rate was quantified accordingly.

### Cell invasion assay

Cell invasion was evaluated using a Transwell assay with 8 μm pore-size membranes precoated with Matrigel. Briefly, after trypsinization, cells were resuspended in serum−free medium and seeded into the upper chamber at a density of 2×10^4^ cells per well. The lower chamber was filled with medium supplemented with 10% (FBS) as a chemoattractant. Following 48 hours of incubation, the cells that had invaded to the lower surface of the membrane were fixed with 4% paraformaldehyde, stained with hematoxylin, and counted under a light microscope.

### Molecular docking analysis

The two-dimensional (2D) structure of 23-HBA was obtained from PubChem (https://pubchem.ncbi.nlm.nih.gov/), and the α-fold structures of key targets were retrieved from the UniProt database (https://www.uniprot.org/). Standard preprocessing of the ligands and receptors was performed using AutoDock Tools (V1.5.6, http://autodock.scripps.edu/) for molecular docking. The binding activity was evaluated based on the docking affinity. Finally, the three-dimensional (3D) conformation of the ligand-receptor complexes was visualized using PyMOL software.

### Western blot analysis

Proteins were extracted from EC9706 and KYSE150 cells treated with 23-HBA using RIPA buffer (Beyotime Biotechnology) supplemented with 1 mmol/L PMSF. Protein lysates were separated by SDS-PAGE and transferred onto PVDF membranes (Bio-Rad, CA, USA). The membranes were blocked with 5% skimmed milk for 1 hour at room temperature, followed by incubation with the following primary antibodies for 3 hours at room temperature: BUB1 (1:1000; abcam, Cat. No. ab195268), STAT3 (1:1000; Cell Signaling Technology, Cat. No. 12640), p-STAT3 (1:1000; Cell Signaling Technology, Cat. No. 9145), CCNB1 (1:1000; Cell Signaling Technology, Cat. No. 12231), CDK1 (1:1000; Cell Signaling Technology, Cat. No. 28439), Bcl-2 (1:1000; Cell Signaling Technology, Cat. No. 15071), Caspase-3 (1:1000; Cell Signaling Technology, Cat. No. 9662), and GAPDH (1:1000; Cell Signaling Technology, Cat. No. 2118). Subsequently, the membranes were incubated with horseradish peroxidase (HRP)-conjugated secondary antibodies for 1 hour at room temperature. Protein signals were visualized using an ECL detection system (ChemiDoc XRS, Bio-Rad, Hercules, CA, USA).

### Xenograft models

Thirty 5-week-old male Balb/c nude mice were obtained from Shandong Pengyue Experimental Animal Technology Co., Ltd. A cell suspension of EC9706 cells at a density of 5×10^6^/mL was prepared, and 100 μL of this suspension was subcutaneously inoculated into the scapular region of each mouse. When the tumor volume reached approximately 100 mm³, the tumor-bearing mice were randomly divided into four groups (n=7 per group) as follows: the blank control group (saline, i.p.), the low-dose 23-HBA group (15 mg/kg, i.p.), the high-dose 23-HBA group (30 mg/kg, i.p.), and the cisplatin group (4 mg/kg, i.p.). All treatments were administered once daily. Tumor volume and body weight were measured every two days. On day 18, all mice were euthanized by anesthesia with sodium pentobarbital (90 mg/kg), and the xenograft tumors were excised and weighed.

### Hematoxylin-eosin staining

Tumor specimens were fixed, embedded in paraffin, and sectioned. The sections were deparaffinized and rehydrated, followed by staining with hematoxylin for 5 minutes and eosin for 5 minutes. After rinsing three times, the sections were dehydrated through a graded ethanol series. Finally, the sections were mounted with neutral resin. Once the resin was fully dried, they were observed and imaged under a microscope at various magnifications.

### Immunohistochemistry

Tumor tissue sections (4 μm thick) were baked, deparaffinized, and subjected to enzymatic antigen retrieval. Subsequently, the sections were incubated overnight at 4 °C with the following primary antibodies: STAT3 (Cell Signaling Technology, Cat. No. 30835), p-STAT3 (Cell Signaling Technology, Cat. No. 9145), and BUB1 (abcam, Cat. No. ab195268). After incubation with the primary antibodies, the sections were treated with a corresponding secondary antibody for 15 minutes at room temperature. Color development was performed using a DAB horseradish peroxidase kit at 37 °C for 15 minutes. The sections were then counterstained with hematoxylin, dehydrated, and mounted. Finally, the stained sections were examined and photographed under a microscope, and the images were analyzed using Aipathwell^®^ software.

### Expression of STAT3, p-STAT3, and BUB1 proteins in tumor tissues

Approximately 0.2 g of tumor tissue was minced on ice, rinsed with ice-cold PBS, and homogenized in 300 μL of RIPA lysis buffer using a tissue homogenizer. The resulting lysate was then incubated on ice for 10 min. The protein samples were separated by SDS-PAGE and transferred onto PVDF membranes (Bio-Rad, CA, USA). The membranes were blocked with 5% skimmed milk at 25 C for 1 hour, followed by incubation with primary antibodies at room temperature for 3 hours. The primary antibodies used were as follows: BUB1 (1:1000; abcam, Cat. No. ab195268), STAT3 (1:1000; Cell Signaling Technology, Cat. No. 12640), p-STAT3 (1:2000; Cell Signaling Technology, Cat. No. 9145), Bcl-2 (1:1000; Cell Signaling Technology, Cat. No. 15071), Caspase-3 (1:1000; Cell Signaling Technology, Cat. No. 9662),and GAPDH (1:1000; Cell Signaling Technology, Cat. No. 2118). Subsequently, the membranes were incubated with horseradish peroxidase (HRP)-conjugated secondary antibodies at 25 °C for 1 hour. Protein bands were visualized using an ECL Western Blotting Detection System (ChemiDoc XRS, Bio-Rad, Hercules, CA, USA).

### Statistical analysis

Data analysis and graphing were performed using GraphPad Prism 8 (GraphPad Software, Inc., USA). Data obtained from three independent experiments are presented as the mean ± standard deviation. Comparisons between two groups were analyzed using the unpaired Student’s *t*-test, while comparisons among multiple groups were analyzed by one-way analysis of variance. All P values < 0.05 were considered statistically significant.

## Results

### Cell proliferation assay

**Figure 1:**
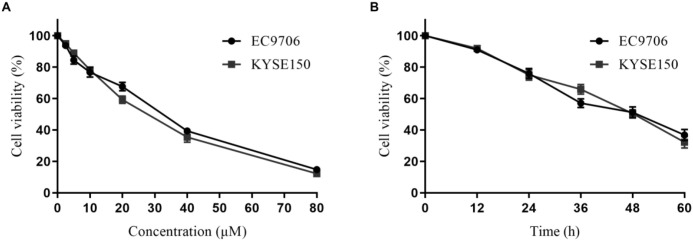
23-HBA suppresses esophageal cancer cell proliferation in dose-and time-dependent manners. **(A)** Dose-response relationship of 23-HBA in esophageal cancer cells after 48 h treatment, *n* = 3. **(B)** Time-response relationship of 23-HBA in esophageal cancer cells, n=3.

The results demonstrated a concentration-dependent enhancement in the inhibitory effects of 23-HBA on the proliferation of esophageal cancer EC9706 and KYSE150 cells (**Figure 1A**). The half-maximal inhibitory concentration (IC_50_) values were determined from standard curves. After 48 hours of exposure, 23-HBA exhibited IC_50_ values of 27.88 μM in EC9706 cells and 24.90 μM in KYSE150 cells, respectively. Based on these IC_50_ values, concentrations of 20 μM and 40 μM were selected for subsequent experiments. Time-course analysis revealed progressively increased inhibition rates when cells were treated with their respective IC_50_ concentrations for durations ranging from 12 to 60 hours (**Figure 1B**). Collectively, these findings indicate that 23-HBA suppresses the proliferation of esophageal cancer cells in both dose- and time-dependent manners.

### Effect of 23-HBA on cell cycle and apoptosis

**Figure 2:**
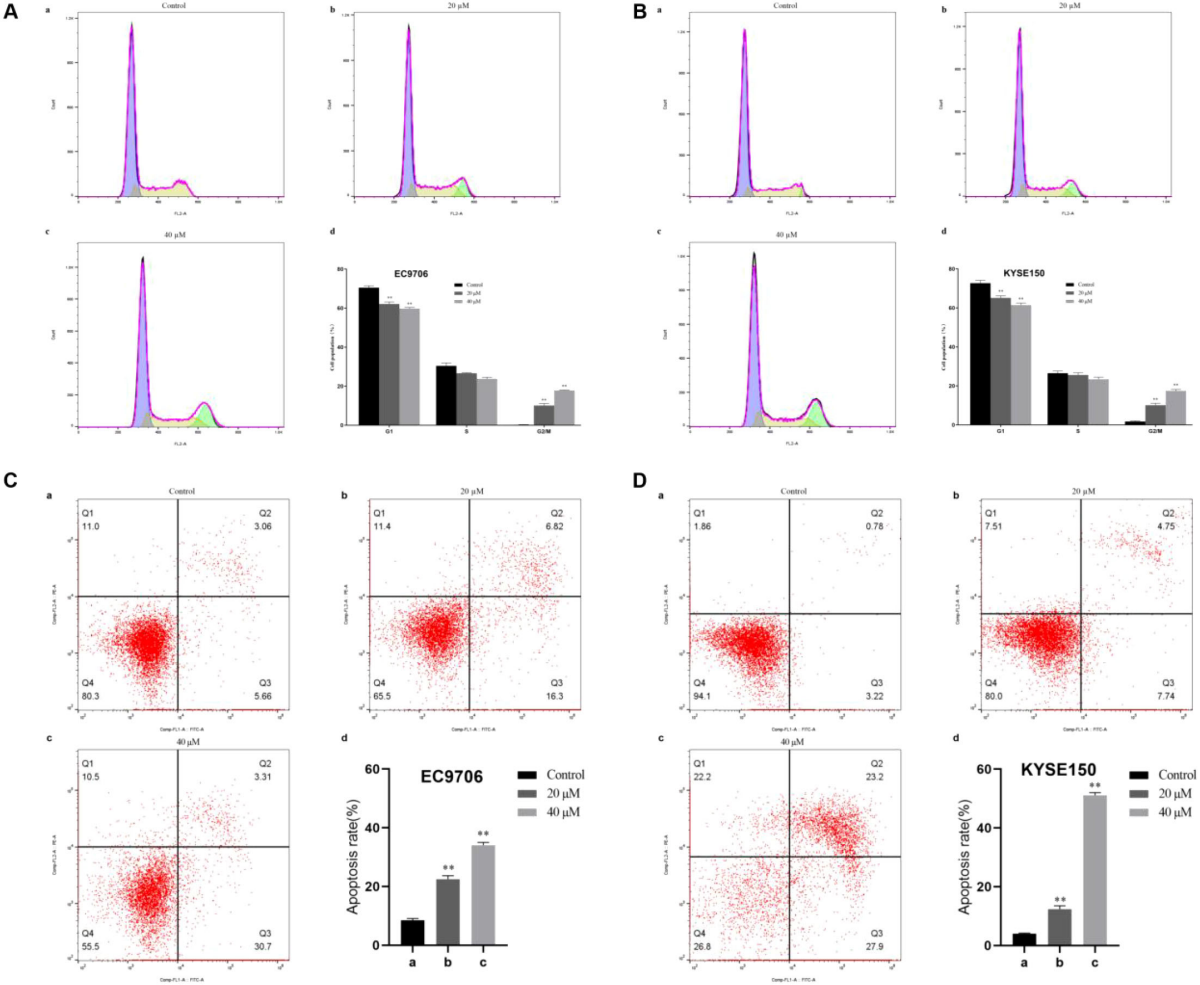
Effect of 23-HBA on the cell cycle and apoptosis of esophageal cancer cells. **(A, B)**: 23-HBA induces G2/M phase arrest in EC9706 and KYSE150 cells, *n* = 3. **p < 0.01 compared with control. **(C, D)**: 23-HBA increases the apoptosis of EC9706 and KYSE150 cells, *n* = 3. **p < 0.01 compared with control.

Cell cycle analysis revealed that treatment with 23-HBA induced significant G_2_/M phase arrest in both EC9706 (**Figure 2A**) and KYSE150 (**Figure 2B**) cell lines, a process potentially regulated by BUB1. Apoptosis analysis demonstrated that 23-HBA treatment effectively induced apoptosis in both EC9706 (**Figure 2C**) and KYSE150 (**Figure 2D**) cells.

### 23-HBA inhibits EC cell migration and invasion

**Figure 3:**
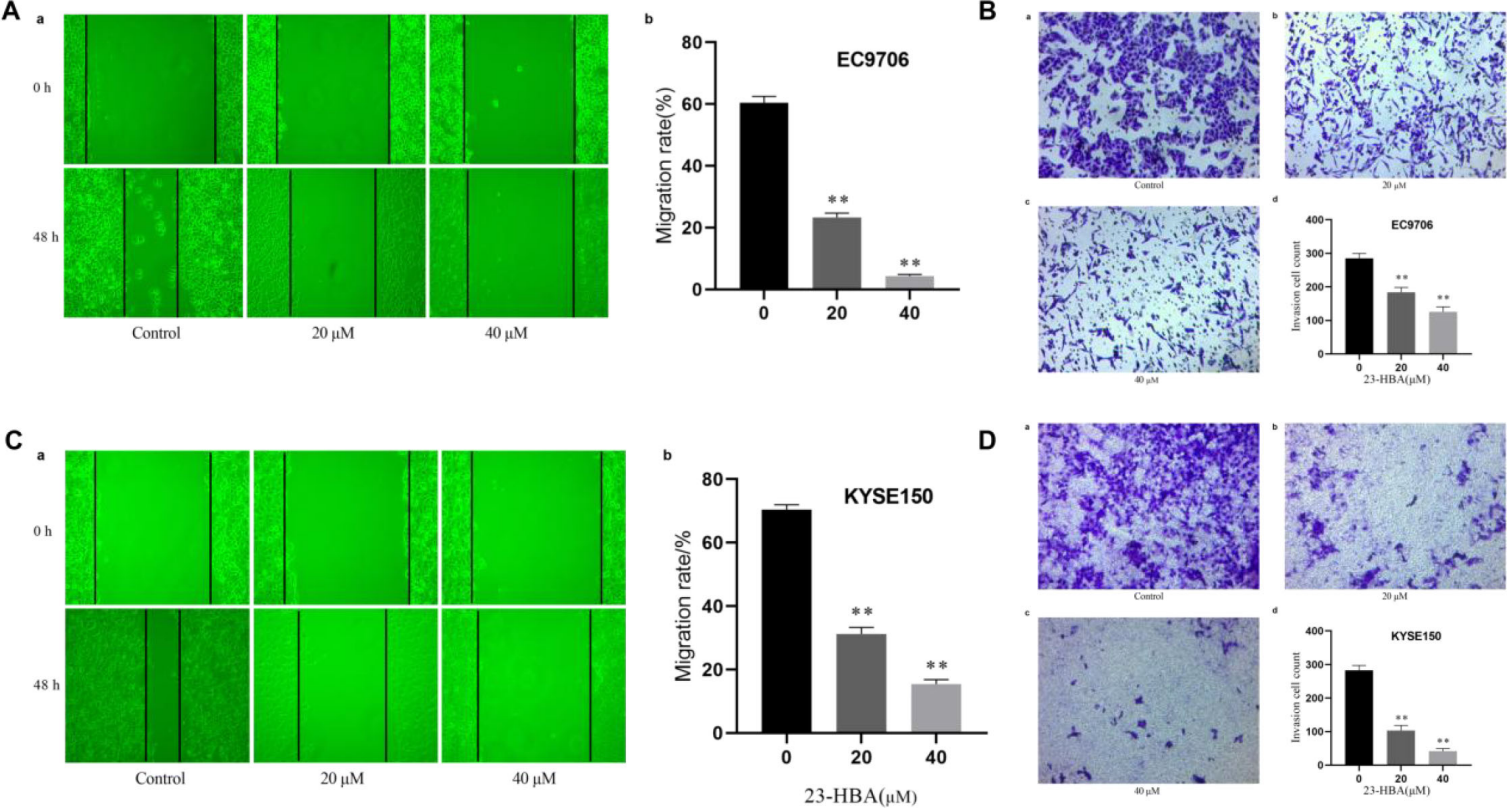
Effect of 23-HBA on migration and invasion of esophageal cancer cells. **(A, C)**: 23-HBA reduces the migration activity of both EC9706 and KYSE150 cells, *n* = 3. **p < 0.01 compared with control. **(B, D)**: 23-HBA reduce the invasion activity of both EC9706 and KYSE150 cell, *n* = 3. **p < 0.01 compared with control.

To investigate the biological significance of 23-HBA in EC, we examined its effects on the migration and invasion of EC cells. Wound healing assay showed that significantly reduced the number of migrating EC9706 and KYSE150 cells (**Figures 3A, C**). Transwell assay with Matrigel showed that 23-HBA led to a significant decrease in the invasive potential of EC9706 and KYSE150 cells (**Figures 3B, D**). In summary, 23-HBA inhibited EC migration and invasion.

### Molecular docking analysis

**Figure 4:**
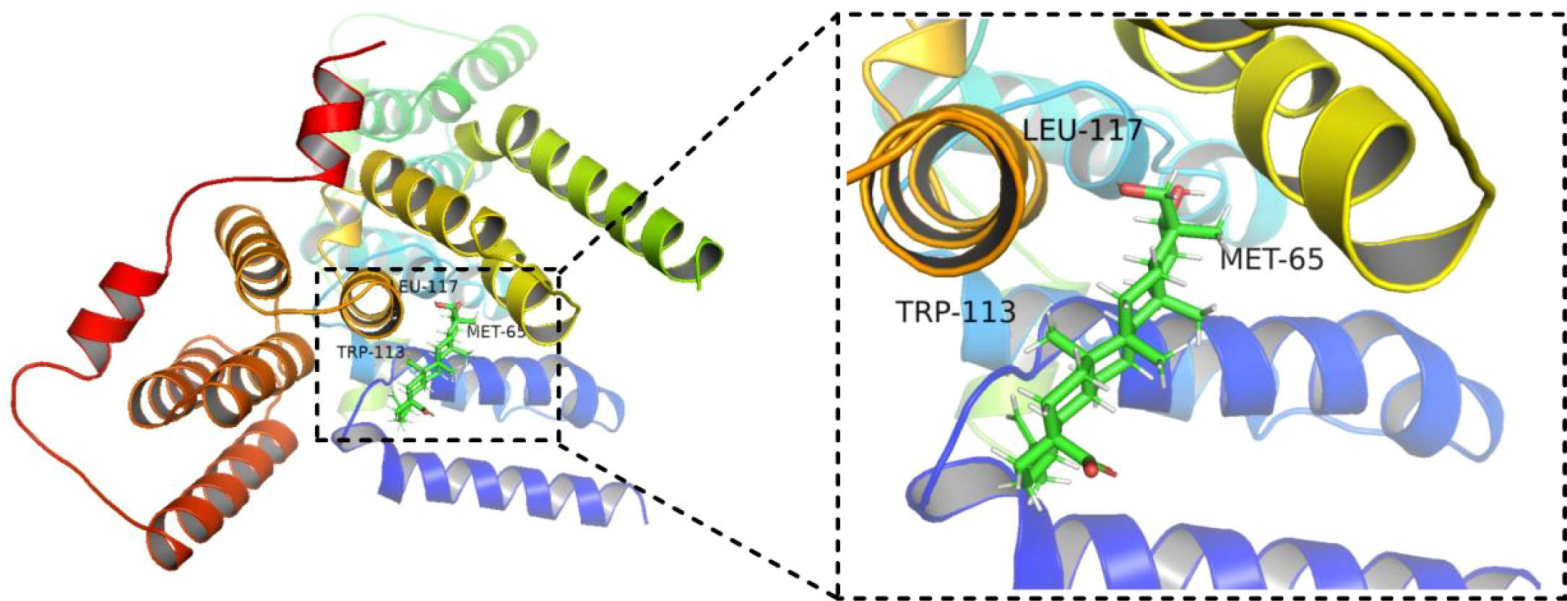
The predicted binding pose of 23-HBA with BUB1. The interaction was simulated by molecular docking and visualized using PyMOL.

Molecular docking was performed between 23-HBA and BUB1 using AutoDockTools 1.5.6, and their binding affinity was calculated. A binding affinity < -4.25 kcal/mol was considered indicative of binding activity between the receptor and ligand, while a value < -5.00 kcal/mol suggested relatively strong binding activity. The results demonstrated that 23-HBA binds to BUB1 with an affinity of -6.2 kcal/mol, reflecting a favorable binding interaction. The docking results were visualized using PyMOL software (**Figure 4**).

### The protein expression levels in different groups were detected by western blotting

**Figure 5:**
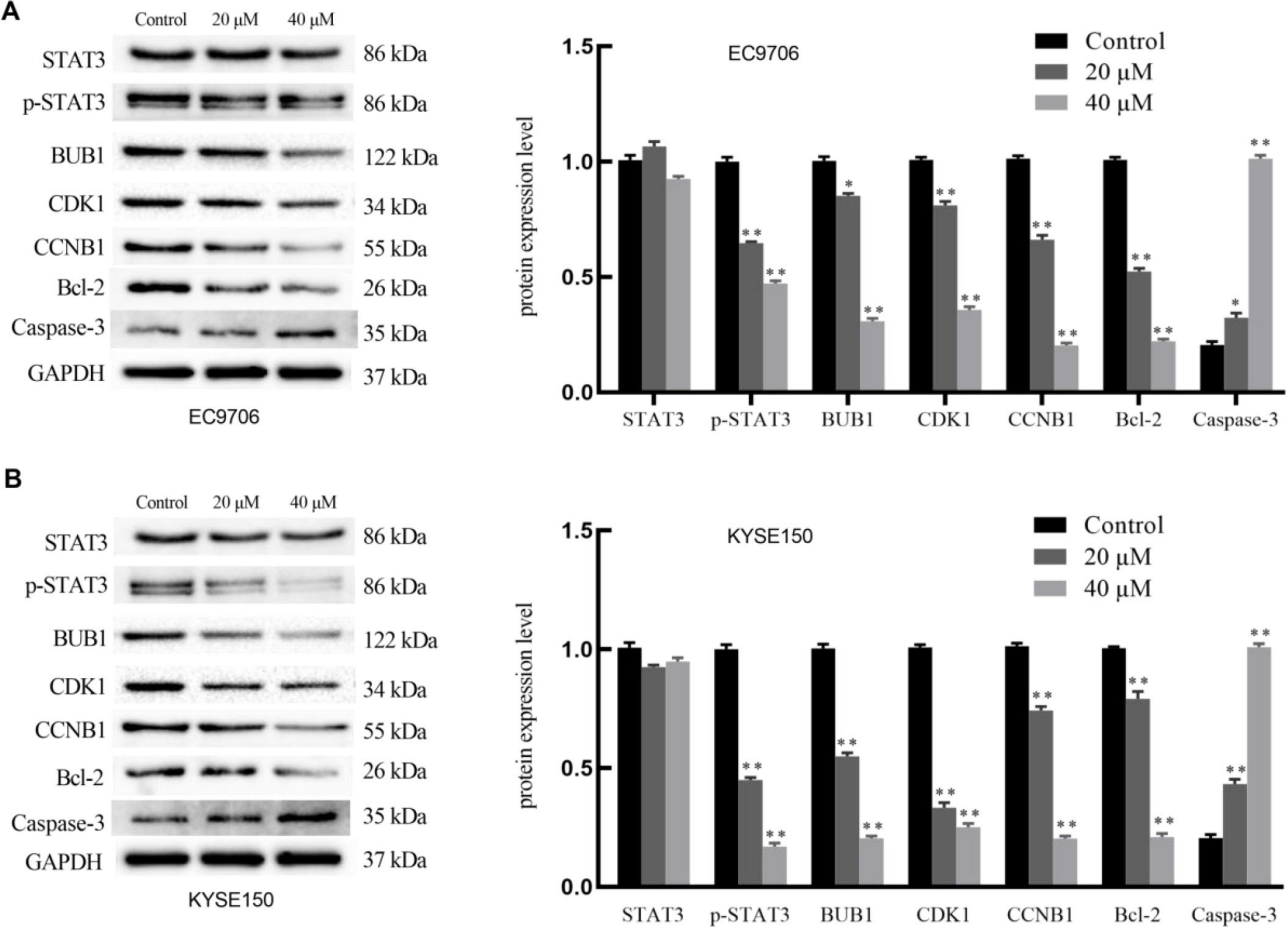
Protein expression of STAT3, p-STAT3, BUB1, CDK1 and CCNB1 in different groups detected using Western blot, *n* = 3. **(A)**: EC9706 cells; **(B)**: KYSE150 cells. *p < 0.05, **p < 0.01 compared with control.

To further investigate the effect of 23-HBA on esophageal cancer cells, we analyzed the expression levels of several key proteins in EC9706 and KYSE150 cells after 48-hour treatment with 23-HBA. The results showed that 23-HBA could inhibit the expression of cell cycle-related proteins CCNB1 and CDK1. 23-HBA significantly downregulated BUB1 and p-STAT3 protein levels, while the total STAT3 protein expression remained largely unchanged. Furthermore, 23-HBA significantly downregulated the protein expression level of Bcl-2 while significantly upregulating that of Caspase-3 (**Figures 5A, B**).

### 23-HBA inhibits tumor growth *in vivo*

**Figure 6:**
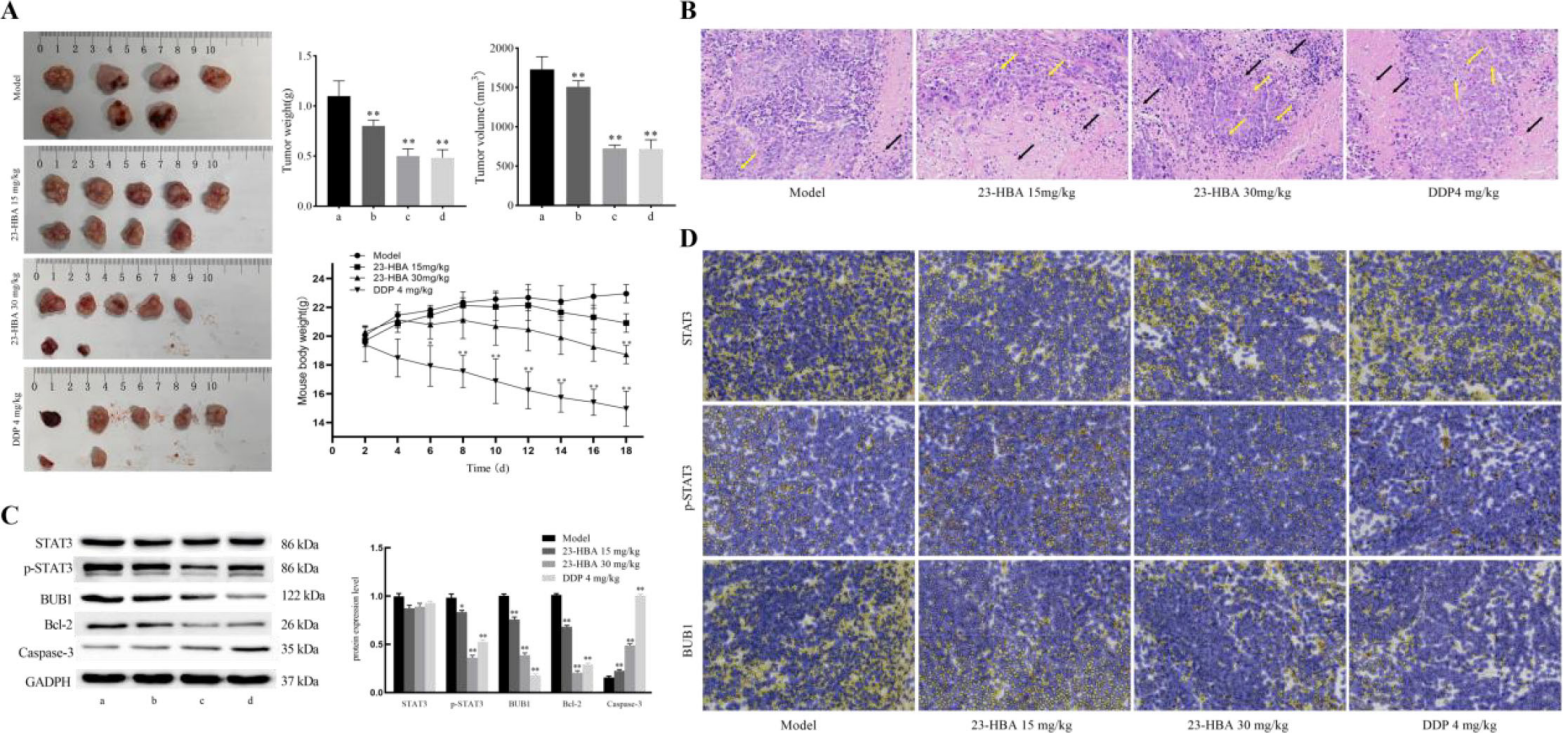
23-HBA inhibits tumor growth *in vivo*. **(A)** 23-HBA significantly reduced tumor volume and weight in nude mice, *n* = 7.*p < 0.05,**p < 0.01 compared with model group. **(B)** Representative images of HE staining showing morphological changes in esophageal carcinoma cells from each group. **(C)** Protein expression levels of STAT3, p-STAT3, BUB1, Bcl-2 and Caspase-3 in tumor tissues detected by western blot, *n* = 3. *p < 0.05,**p < 0.01 compared with model group. **(D)** localization and positive expression of STAT3, p-STAT3, and BUB1 proteins detected by immunohistochemistry.a: model; b: 23-HBA 15 mg/kg; c: 23-HBA 30 mg/kg; d: DDP 4 mg/kg.

To investigate the effect of 23-HBA on tumor formation *in vivo*, we established a xenograft tumor model in nude mice using esophageal cancer cells. The results showed that administration of 23-HBA at doses of 15 mg/kg and 30 mg/kg significantly reduced both tumor volume and tumor weight in the mice (**Figure 6A**). In addition, throughout the experimental period, mice in the 23-HBA groups exhibited stable body weight with no significant difference compared with the model group, indicating that 23-HBA has low toxicity and is well tolerated at the administered doses.(**Figure 6A**). HE staining analysis demonstrated distinct morphological alterations in the treatment group compared to the model group. Tumor cells exhibited irregular contours, decreased density, an elevated nuclear-to-cytoplasmic ratio, and nuclear atypia, with infrequent mitotic figures (yellow arrows). Extensive areas of tumor necrosis were evident (black arrows), characterized by nuclear pyknosis, karyorrhexis, or karyolysis, accompanied by cytoplasmic degradation. Notably, significant inflammatory infiltration was absent in these regions (**Figure 6B**). As shown by Western blot (**Figure 6C**) and immunohistochemistry (**Figure 6D**), 23-HBA significantly reduced the protein levels of BUB1 and p-STAT3 compared with the model group, whereas the expression of total STAT3 protein showed no significant change. Furthermore, Western blot analysis revealed that 23-HBA significantly downregulated the protein expression level of Bcl-2 while significantly upregulating that of Caspase-3.

## Discussion

Botanically derived Chinese herbal medicines have been widely used in Traditional Chinese Medicine (TCM). Long-term clinical practice has revealed that some of these natural compounds play significant roles in cancer treatment (18). The herb *Pulsatilla chinensis* (Baitouweng) was first documented in the Shennong Ben Cao Jing (Divine Farmer’s Materia Medica). It is traditionally used to clear damp-heat and resolve blood stasis. The pathogenesis of esophageal cancer primarily involves deficiency, stasis, phlegm, and fire. During its progression, these factors often lead to fire transformation from qi stagnation and obstruction by phlegm-stasis, which may manifest as intense heat-toxicity. This pathological mechanism partially overlaps with the indications for Pulsatilla chinensis, providing a theoretical basis for its potential application in treating esophageal cancer (19). Triterpenoid saponins are the well-established primary active constituents of *Pulsatilla chinensis* (Baitouweng) and have been extensively studied. Among them, 23-hydroxybetulinic acid (23-HBA) is a major representative triterpenoid saponin isolated from this plant (20). Previous studies have indicated that, in addition to inducing cancer cell apoptosis, 23-HBA can enhance the efficacy of certain anti-tumor drugs (21). In the present study, we found that 23-HBA inhibits the viability of both EC9706 and KYSE150 esophageal cancer cell lines in a dose- and time-dependent manner.

BUB1 is a mitotic checkpoint serine/threonine kinase that serves as a critical component of the spindle assembly checkpoint (SAC). Dysfunction of the SAC, caused by genetic mutations or aberrant expression of BUB1, can lead to chromosomal aneuploidy, which is associated with the development and progression of various human malignancies (22). Currently, various alterations of the BUB1 gene have been identified in multiple cancer tissues and cell lines, encompassing genetic mutations such as deletions and point mutations, as well as aberrant expression at both the transcriptional and protein levels (23). BUB1 mediates cell death and suppresses spontaneous tumorigenesis. It is also implicated in the DNA damage response, and mutations in this gene are associated with aneuploidy (24). In our study, 23-HBA downregulated BUB1 expression. Reduced BUB1 expression has been shown to inhibit tumor cell proliferation, invasion, and migration, while also decreasing cell viability and promoting apoptosis (25, 26). The growth-inhibitory effect of 23-HBA on esophageal cancer cells via BUB1 suppression may be associated with the downregulation of the STAT3 signaling pathway (27). Cell division is accomplished through the cell cycle. However, dysregulated cell division can lead to uncontrolled proliferation, ultimately culminating in the hallmark of cancer (28). Our results demonstrate that 23-HBA induces G2/M phase arrest in cancer cells. This finding is mechanistically significant as BUB1, CCNB1, and CDK1 are crucial regulators governing the G2/M to M phase transition. Specifically, BUB1 promotes cancer cell proliferation by accelerating the G2/M transition (29), while the CCNB1/CDK1 complex drives this transition by providing essential bioenergy through phosphorylation, and its reduction leads to G2/M arrest (30). Furthermore, clinical evidence indicates that overexpression of both BUB1 and CDK1 correlates with tumor aggressiveness and poor patient survival (31). Our study demonstrated that 23-HBA inhibits esophageal cancer cell proliferation by downregulating BUB1, CCNB1, and CDK1, thereby blocking the G2/M to M phase transition. These results align with prior research (32, 33).

The STAT3 signaling pathway functions as a signal transducer and transcription activator. Research has demonstrated that STAT3 is aberrantly activated in various malignant tumors and is implicated in key cancer-related biological processes including proliferation, apoptosis, and metastasis (34, 35). Importantly, elevated p-STAT3 expression is a critical factor driving malignant phenotypes such as migration and invasion in esophageal cancer. Suppressing the STAT3 signaling pathway significantly suppresses these malignant phenotypes (36, 37), thus establishing STAT3 as a promising therapeutic target for esophageal cancer treatment. STAT3 was identified as a substrate of BUB1, and BUB1 is critical for STAT3 phosphorylation (16). We found that 23-HBA reduced p-STAT3 expression levels in esophageal cancer EC9706 and KYSE150 cells without affecting total STAT3 protein levels. This suggests that 23-HBA may effectively inhibit STAT3 phosphorylation and STAT3-mediated transactivation in esophageal cancer cells by downregulating BUB1. Current studies have also identified elevated STAT3 phosphorylation levels in gliomas, lung cancer, and endometrial carcinoma (38–40). Currently, targeted tumor therapy through direct or indirect blockade of STAT3 (41–43) has emerged as a promising therapeutic strategy, making STAT3 blockade a new direction in cancer treatment.

## Conclusion

In summary, our study demonstrates that 23-HBA suppresses proliferation, migration, and invasion of esophageal cancer cells, disrupts cell cycle progression, induces apoptosis, and reduces xenograft tumor growth. The anti-tumor effects are potentially mediated through the BUB1/STAT3 signaling pathway, providing new insights for targeted therapy development in esophageal cancer.

## Data Availability

The raw data supporting the conclusions of this article will be made available by the authors, without undue reservation.
